# Vitamin D: Link between Osteoporosis, Obesity, and Diabetes?

**DOI:** 10.3390/ijms15046569

**Published:** 2014-04-17

**Authors:** Flávia Galvão Cândido, Josefina Bressan

**Affiliations:** Laboratory of Body Composition and Energy Metabolism, Departamento de Nutrição e Saúde-CCB II, Universidade Federal de Viçosa, Avenida PH Rolfs, s/n, Viçosa, MG 36570-900, Brazil; E-Mail: ppgcnut@ufv.br

**Keywords:** vitamin D, osteoporosis, obesity, diabetes mellitus, PTH, bone mineral density, falls, fractures, osteoblasts, osteoclasts

## Abstract

Vitamin D (1,25(OH)_2_D_3_) is a steroid hormone that has a range of physiological functions in skeletal and nonskeletal tissues, and can contribute to prevent and/or treat osteoporosis, obesity, and Type 2 diabetes mellitus (T2DM). In bone metabolism, vitamin D increases the plasma levels of calcium and phosphorus, regulates osteoblast and osteoclast the activity, and combats PTH hypersecretion, promoting bone formation and preventing/treating osteoporosis. This evidence is supported by most clinical studies, especially those that have included calcium and assessed the effects of vitamin D doses (≥800 IU/day) on bone mineral density. However, annual megadoses should be avoided as they impair bone health. Recent findings suggest that low serum vitamin D is the consequence (not the cause) of obesity and the results from randomized double-blind clinical trials are still scarce and inconclusive to establish the relationship between vitamin D, obesity, and T2DM. Nevertheless, there is evidence that vitamin D inhibits fat accumulation, increases insulin synthesis and preserves pancreatic islet cells, decreases insulin resistance and reduces hunger, favoring obesity and T2DM control. To date, there is not enough scientific evidence to support the use of vitamin D as a pathway to prevent and/or treat obesity and T2DM.

## 1. Introduction

Vitamin D is a topic of great interest for the scientific community as well as for the layman. The commonly-know function of vitamin D has been associated with skeletal tissue, in which vitamin D influences mineralization, bone turnover rate, and occurrence of fractures, contributing to the prevention and treatment of osteoporosis [1]. Nevertheless, the recent discovery of ample distribution of vitamin D receptors (VDR) in non-skeletal tissues dramatically increased the interest in this vitamin as a therapeutic modality for the prevention of chronic diseases, such as obesity and Type 2 diabetes mellitus (T2DM) [2]. During the two-year period of 2012 and 2013, over 350 articles were published in high impact journals containing vitamin D and obesity/diabetes in the title. Additionally, factors derived from skeletal tissue, such as the osteocalin and the osteopontin, can affect body weight and glycemia [3,4], suggesting an indirect role of vitamin D in these parameters.

Despite the relevance of the topic and the evidence of the association between vitamin D, obesity, and T2DM, current recommendations of this vitamin only considered the effects of vitamin D on skeletal tissue [5–7]. In the present review, we critically investigated the effects of vitamin D on osteoporosis, obesity, and T2DM, with special focus on randomized controlled trials conducted on adults and the elderly. We intended to evaluate vitamin D as a link between skeletal and chronic non-skeletal disorders and not to exhaustively revisit the topic. The interrelation between osteoporosis, obesity, and T2DM and their mechanisms are also considered, regarding the effects of vitamin D on these relationships.

## 2. Methodology

Medline/PubMed, Science Direct, Scientific Electronic Library Online (SCIELO), and Latin American and Caribbean Health Sciences Literature-LILACS electronic databases were searched to identify studies published within the last 10 years regarding the effects of vitamin D on osteoporosis, obesity, and diabetes. For data searches, the following main terms were used alone or in association: vitamin D, vitamin D receptor, parathormone (PTH), osteoporosis, bone health, obesity, adiposity, weight, insulin resistance, diabetes, metabolic syndrome, and β-cell function. Review and original articles were selected according to their titles and abstracts. Each selected manuscript was then studied critically.

## 3. Vitamin D

Vitamin D, in its metabolically active form, 1,25(OH)_2_D_3_, is a steroid hormone obtained after hepatic (C-25 position), and not exclusively, kidney (C-1) hydroxylations. Its precursors can be acquired from the diet as well as sun exposure; the latter being due to non-enzymatic reactions by exposing the skin to ultraviolet radiation [8]. Sun exposure can produce more vitamin D than the diet, even when nutritional supplements of the recommended concentrations are included [3]. However, the large number of factors affecting the synthesis and/or bioavailability of vitamin D and the fact that ultraviolet radiation is classified as a Group 1 carcinogen by the International Agency for Research on Cancer, increase the importance of diet in maintaining adequate levels of this vitamin [9].

Decreased sun exposure related to latitude, seasonality, time of day, atmospheric components, clothing, and sunscreen use, compromise vitamin D synthesis in individuals [10]. The pigmentation of the skin also affects vitamin D synthesis capacity, since melanin effectively absorbs electromagnetic radiation and competes with the vitamin’s precursors [11]. The bioavailability of vitamin D depends on its intestinal absorption capacity, liver health of the individuals, and their fat storage. Adipose tissue easily absorbs vitamin D ingested or produced by chemical affinity. Some authors suggest that the accumulation of vitamin D in adipose tissue is important for its subsequent release during times of reduced production (for example, during winter when the fat storage decreases) [12]. All of these factors should be taken into consideration upon evaluating studies involving the role of vitamin D.

The 25(OH)D_3_ is considered the best indicator of nutritional and functional status of vitamin D, because it is present in higher concentrations in the blood, has a long half-life (15 to 20 days), and there are many available techniques for its evaluation [13]. Although 1,25(OH)_2_D_3_ corresponds to the active form of the vitamin, it circulates in the bloodstream in concentrations 1000 times lower to that of 25(OH)D_3_ and its half life is only 4 to 6 h [14]. In addition, levels of 25(OH)D_3_ fail as indicators of 1,25(OH)_2_D_3_ status just in patients with calcitriol synthesis abnormalities (e.g., sarcoidosis) or with rare phosphate or vitamin D metabolism disorders [2,13]. The most accepted classification of vitamin D status considers deficient those individuals who have 25(OH)D_3_ serum levels below 20 ng/mL (50 nmol/L), insufficient in those with levels between 21 to 29 ng/mL (52.5 to 72.5 nmol/L), and sufficient with levels of 30 to 44 ng/mL (75 to 110 nmol/L) [2,15,16]. Although there are efforts to raise these normality values, Bischoff-Ferrari *et al*. [16] stressed that there is currently no data to support that levels above 50 ng/mL result in more additional benefits than the 30 to 44 ng/mL range. The benchmark, 100 ng/mL (250 nmol/L), should be considered as a safety limit, but not as an upper limit to reach in clinical practice. Although this level is considered safe, it is not the desirable level for optimal health results [15].

**Figure 1.**
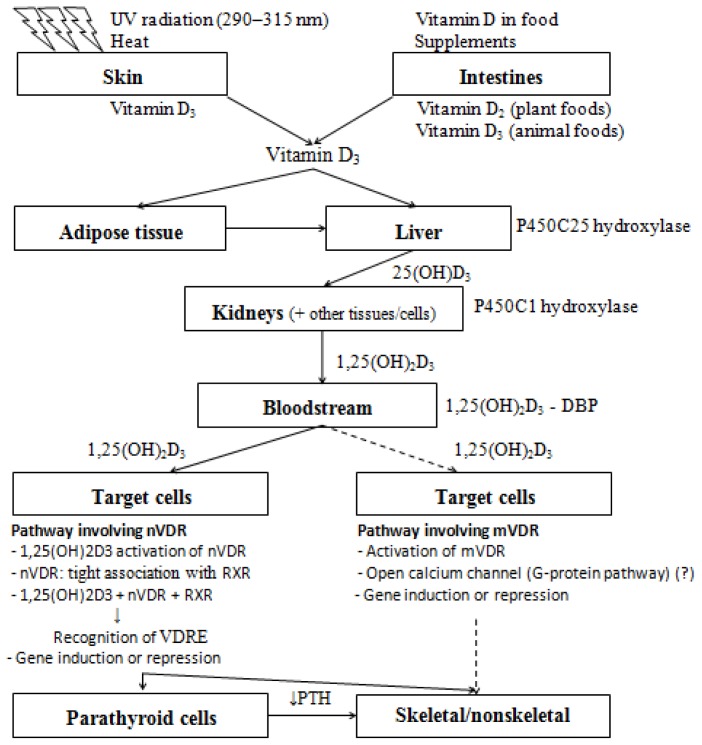
Vitamin D biosynthesis and action. Precursors of vitamin D are incorporated by foods, supplements, or synthesized by skin after UV-radiation and heat. These precursors are stocked in adipose tissue or carried to liver and kidneys for hydroxylation by the enzymes P450C25 and P450C1 hydroxylases, respectively. The metabolic active form of vitamin D (1,25(OH)_2_D_3_) is transported through the bloodstream by vitamin-D-binding protein (DBP). In target cells, 1,25(OH)_2_D_3_ follows two distinct pathways. Target cells that have nuclear vitamin D receptors (nVDR) will trigger a better understood pathway involving: activation of nVDR by 1,25(OH)_2_D_3_; connection with other transcription factors, such as retinoid X receptors (RXR); formation of a complex capable of recognizing response elements of vitamin D (VDRE); and induction or repression of specific genes. On the other hand, target cells that have membrane vitamin D receptors (mVDR) will trigger a less understood pathway, maybe involving the activation of mVDR by 1,25(OH)_2_D_3_ and the rapid opening of a G-protein-coupled membrane-bound calcium channel. These mechanisms modulate the synthesis of parathormone (PTH) and, together with the diminished PTH levels, act in the metabolism of skeletal and non-skeletal tissues. Adapted with permission from Nature Publishing Group: Nature Reviews Cancer [18] copyright 2003.

The active vitamin D is capable of binding to an intracellular transcription receptor called vitamin D receptor (VDR). The identification and cloning of VDR occurred only in 1987 and, since then, new tissue-specific functions of vitamin D have been discovered. Currently, it is known that the VDR is widely distributed among tissues and that the absence of the receptor is the exception, not the rule [5]. The majority, if not all of vitamin D’s functions, are mediated by the VDR acting in the regulation of gene expression in specific DNA regions. Vitamin D binds to its nuclear receptor (nVDR) with high affinity and specificity; the receptor works in partnership with other transcription factors, such as the retinoid X receptor (RXR). The group formed by vitamin D, RXR, and VDR can recognize the vitamin D responsive elements (VDREs) of genes regulated by vitamin D [8]. Some immediate vitamin D action may occur by other less-known pathways, which involves the participation of the VDR bound to the plasma membrane (mVDR) and not the nVDR (Figure 1) [5].

One of the most common disorders related to low vitamin D levels is secondary hyperparathyroidism. Parathyroid cells that have VDR and vitamin D deficiency, with or without low expression of VDR in these cells, causes an increase in circulating levels of the parathyroid hormone (PTH), which is responsible for a number of metabolic alterations in skeletal and non-skeletal tissues [17]. Therefore, vitamin D mediates metabolic alterations resulting from high PTH levels.

## 4. Vitamin D and Osteoporosis

### 4.1. Mechanisms of Action

The main role of vitamin D in bone metabolism is to increase the plasma levels of calcium and phosphorus, essential for mineralization. The increase in circulating levels of calcium is also necessary for the proper functioning of nerve transmission, neuromuscular junctions and hormone secretion, in particular PTH [1]. All these mechanisms increase bone mineral density and reduce the risk of falling by increasing muscle tone, which contributes to reducing osteoporosis and its consequences.

Vitamin D in its active form (1,25(OH)_2_D_3_) is able to increase circulating levels of calcium and phosphorus to normal levels through three pathways. The first pathway, and most well established, is by stimulating the absorption of calcium and phosphate in the intestine, particularly in the duodenum and jejunum. This occurs due to the opening of calcium channels and by the formation of calcium-binding protein, independent of PTH [1,4]. Vitamin D is able to increase the absorption rate of calcium in the intestine, which is usually 10% to 15% during passive transport, 30% to 40% under normal conditions, reaching up to 60% to 80% during pregnancy and lactation [14,19]. The second pathway, dependent on PTH, occurs through mobilization of calcium and phosphorus from bone. In this process, there is increased expression of the receptor activator for nuclear factor κB ligand (RANKL) protein in the osteoblasts, capable of binding to the pre-osteoclast RANK and promoting osteoclastogenesis and bone resorption [14,20,21]. Vitamin D in the osteoblasts is also capable of highly stimulating the synthesis of osteocalcin and moderately osteopontin [22]; two structural proteins present in the organic matrix related to bone remodeling that have a hormonal function in peripheral tissues [6,7]. In osteoclasts, vitamin D exerts a direct function by stimulating osteoclastogenesis [23], although the indirect action via osteoblasts is the most recognised. The third pathway is also dependent on the PTH and involves the increase in renal retention of calcium due to increased tubular reabsorption or a decrease of filtered load [19]. The renal function of vitamin D is well known and many proteins involved in the process have been identified, although the molecular mechanisms are not well understood [1].

**Figure 2.**
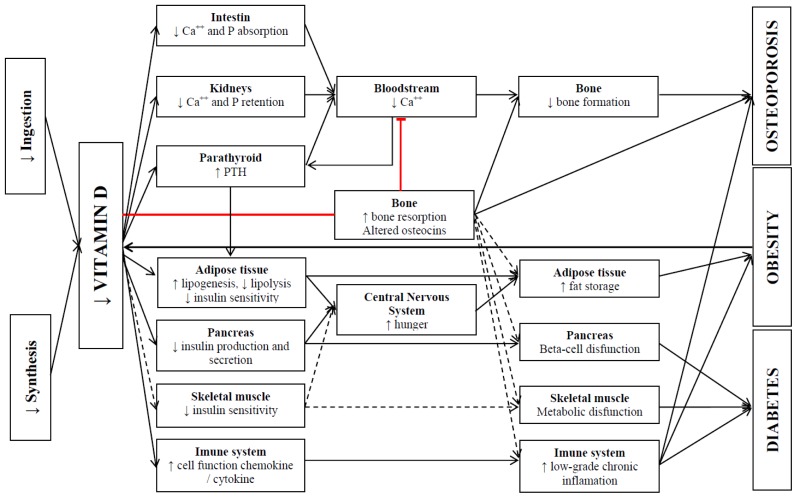
A simplified model of potential mechanisms in the modulation of osteoporosis, obesity, and diabetes through reduction in vitamin D. Red lines: inhibition pathways. Dotted lines: not well established mechanisms. PHT: parathormone; Ca^++^: calcium; p: phosphorus.

Mineralization is a passive process, but it only occurs when calcium and vitamin D are available in sufficient quantities. In vitamin D deficiency, there is a decrease in circulating levels of calcium and increased PTH levels. PTH acts by increasing P450C1 hydroxylase activity in the kidney, which consequently increases vitamin D serum levels, and is a potent agent in bone resorption. In this new phase, the circulating levels of vitamin D and calcium are normal, but the bone reserves become compromised. If vitamin D deficiency occurs for a prolonged period, substrates for synthesis of the active form of the vitamin may be reduced and the resulting bone loss can lead to osteoporosis [4,14]. In contrast, normal vitamin D levels promote adequate calcium levels in the bloodstream. The parathyroid gland cells are sensitive to these two elements by having VDR and calcium-sensing receptors, which act by combating PTH hypersecretion and the resulting bone resorption [17,24]. It is worth remembering that bone tissue is dynamic and that the resorption process is also part of the formation process. Bone loss occurs only when there is an imbalance, with increased resorption in relation to formation [25]. Vitamin D, although it may act on bone resorption, promotes bone formation over the long term [26], in part by increasing intestinal absorption of calcium and combating the hypersecretion of the potent bone resorption agent, PTH (Figure 2).

### 4.2. Scientific Evidences

The state of vitamin D is related to bone mineral density (BMD) in both deficient as well as insufficient individuals. Cross-sectional studies have demonstrated a positive relationship between serum levels of 25(OH)D_3_ and BMD [27–29] with varying limits of 25(OH)D_3_, from which the BMD reaches a plateau. These limits vary from 20 ng/mL (50 nmol/L) [28] to 36 ng/mL (90 nmol/L) [27], depending on the target population and the geographical region where BMD was measured. Similarly, a decrease was observed in the incidence of fractures in individuals with 25(OH)D_3_ serum levels greater than 12 ng/mL (30 nmol/L) [30] and the average mean 25(OH)D_3_ levels were higher in subjects with less severe fractures compared to those with severe fractures [31]. It is noteworthy that these reference values seem to be the same at which PTH level stabilization occurs [32], *i.e*., 25(OH)D_3_ levels sufficient to establish the best relationship with BMD and with fracture risks are also responsible for the lower levels of PTH, suggesting an important role of PTH in the process.

The effect of vitamin D supplementation, concomitant or not with calcium, has been evaluated in various controlled clinical trials (Table 1). As primary outcomes, the studies had BMD and/or the incidence of falls/fractures, and the fractures and BMD were evaluated in different anatomical regions. Most of them involved postmenopausal women and/or those with high risk of developing fractures. The daily doses of calcium adopted in the studies ranged from 500 to 1200 mg/day. Vitamin D, in turn, had wide variation in dosage, form and frequency of administration.

#### 4.2.1. Vitamin D and BMD

Among the studies that evaluated the effects of vitamin D on BMD [26,33–45], only four [37,39–41] showed no significant effect of supplementation, validating the use of vitamin D as a way to prevent and/or treat bone loss. These four studies [37,39–41], however, included higher doses of vitamin D (2857 to 7143 IU/day) [37,39,40], younger men and women with reduced risks of having bone formation deficiency [37,40], less time duration [39,41], and/or small populations [39–41]. All these factors may have contributed to the lack of significant results in these studies.

The use of moderate doses of vitamin D (800 IU/day) appears to be more effective in the reduction of bone turnover and increased BMD compared to high doses (6500 IU/day) [42]. However, in obese subjects, the 7000 IU/day dose of this vitamin was effective in reducing bone turnover and increasing BMD [45]. It is possible that the uptake of vitamin D from adipose tissue is responsible for the increase in the action threshold of vitamin D in bone health in overweight subjects. Additionally, vitamin D supplementation appears to have different effects on individuals with different VDR polymorphisms. Black women with genotype FF for the Fokl allelic region had higher bone loss, but were more responsive to vitamin D supplementation [43]. This information indicates that the population characteristics influence the results of vitamin D supplementation on BMD. It is noteworthy that the best anatomical region for BMD monitoring is the posteroanterior spine region, having greater precision and being more responsive to treatment [46]. The use of other anatomical regions may not be as sensitive and may impair the analysis of the results.

#### 4.2.2. Vitamin D and Falls/Fractures

Among the studies that evaluated the effects of vitamin D on the risk of falls and/or fractures [33,34,47–56], positive effects were observed in some [33,47,48,51,54], but not all [34,49,50,52,53,55,56], studies and in two of them [53,55] supplementation was harmful. The improvement in the evaluated parameters showed up to a 52% decrease in the risk of fall [33] and a 33% decrease in the risk of fractures in the hip, wrist/forearm, and vertebrae [47].

The lack of significant results in some studies may have various causes. It is possible that the falls/fractures are less sensitive parameters than BMD to assess the effects of vitamin D supplementation, since they occur at a more advanced stage of bone depletion, and thus some studies might not have been able to demonstrate the effect of the vitamin on these parameters. Some studies showed less than 60% compatibility in the intake of supplements, vitamin D, and calcium [49,50] and that the follow-up treatment is essential to verify the success of any intervention. Furthermore, the 400 IU/day dose of vitamin D is insufficient to raise and maintain serum levels within the normal range and to decrease the risk of hip and other nonvertebral fractures [44,57]. Bischoff-Ferrari *et al*. [58] suggest that a minimum daily dose of 800 IU is needed to affect the incidence of fractures.

Although vitamin D has a protective role in the incidence of falls/fractures, proven by most studies, the deleterious effect of high dosages should be noted. Sanders *et al*. [55] showed that annual mega doses of 500,000 IU of vitamin D increase the risk of falls and fractures by 15% and 26%, respectively. The authors speculate that high serum levels of vitamin D or its metabolites, resulting from the large annual dose, and subsequent decrease in these levels, or both, may be the cause of harm. Smith *et al*. [53] also found a deleterious effect of a 300,000 IU annual dose of vitamin D on the incidence of hip fracture (49% increase). It is noteworthy, however, that both aforementioned studies [53,55] did not ally the consumption of calcium with the vitamin D supplementation. The study conducted by Harwood *et al*. [33] employed the same dose and dosing interval as that of Smith *et al*. [53], but included 1000 mg/d of calcium and obtained positive results. Another question concerns the dosing interval. Trivedi *et al*. [47] adopted the same dosage as Smith *et al*. [53], but in a rationed manner (100,000 IU every four months) and obtained positive results with vitamin D, even when not combined with calcium. These studies indicate that the addition of calcium and the adoption of shorter intervals between administrations can be essential to the success of vitamin D supplementation in the prevention and/or control of osteoporosis.

## 5. Vitamin D, Obesity, and T2DM

### 5.1. Mechanisms of Action

The role of vitamin D in the pathophysiology of obesity and T2DM is a subject of debate in the scientific community. Although many observational studies have demonstrated a negative association between indicators of obesity and/or T2DM and serum levels of vitamin D, the cause and effect relationship of these variables is not well established [59].

Some mechanisms are proposed to explain how vitamin D deficiency promotes obesity and T2DM. The VDRs are sensitive to 1,25(OH)_2_D_3_ and widely expressed in adipose, pancreatic, and possibly muscle cells [60,61]. Adipocytes and β-pancreatic cells also possess the capacity to activate vitamin D by having the enzyme 25-hydroxyvitamin D 1-α-hydroxylase [62,63]. In adipocytes, there is evidence that vitamin D inhibits the active form of adipogenic transcription factors and fat accumulation during the differentiation phase [63]. In the β-pancreatic cells, vitamin D appears to modulate insulin synthesis via the nVDR, since there are VDREs in the insulin promoter genes [64]. Vitamin D may also promote morphological improvement in pancreatic islet cells, decrease apoptosis, and have nongenomic effects mediated by mVDR [65]. In skeletal muscle, vitamin D can decrease insulin resistance and increase glucose uptake [66]. The increased secretion and/or insulin action is related to decreased hunger and food intake, helping to reduce obesity [67]. Additionally, mice with VDR deficiency in adipocytes presented a lean phenotype [68]; 1,25(OH)_2_D_3_ directly activates the human insulin receptor gene [69], the peroxisome proliferator-activated receptor delta (PPAR-δ) [70], besides stimulating the insulin receptor expression and increasing the transport of *in vitro* insulin-mediated glucose [71]. Thus, vitamin D deficiency can cause the accumulation of body fat and compromise normal metabolic functioning, favoring obesity and T2DM.

Adipose tissue, in turn, can act by lowering serum levels of vitamin D. This fat soluble vitamin can be stored in adipose tissue and further questions still remain about how its reentry into the bloodstream occurs [72]. Thus, low levels of serum vitamin D would be a result of obesity and not its cause. This hypothesis gained prominence recently with the publication of a study that assessed the causality and direction of the relationship between BMI and serum 25(OH)D_3_ [73]. After gathering and evaluating consistent data from 21 cohorts (over 42,000 participants), the authors concluded that elevated BMI levels are the cause of the decrease in 25(OH)D_3_ in the bloodstream. Regardless of the source, however, decreased levels of serum vitamin D can reduce circulating calcium and induce secondary hyperparathyroidism [17]. The increase in PTH may induce weight gain, obesity, and T2DM. The PTH chronically increases intracellular levels of ionic calcium in adipocytes, which may act reciprocally on increased expression of fatty acid synthase (FAS)—a key regulatory enzyme in the deposition of lipids—and on decreased lipolysis. Decreased thermogenesis and lipid oxidation through the down-regulation of uncoupling proteins is also suggested [67].

Vitamin D can also act indirectly on the control of obesity and diabetes. The high increase in osteocalcin synthesis by osteoblasts appears to regulate body fat and improve glucose tolerance by stimulating insulin synthesis in the pancreas and adiponectin in adipocytes [74]. On the other hand, increased osteopontin, even if moderate, stimulates the growth of adipose tissue and the development of chronic low-grade inflammation associated with obesity and insulin resistance [75,76]. Despite conflicting mechanisms, the overall modulation of the osteocalcins by vitamin D seems to favor the control of obesity and its comorbidities [77]. Other targets of vitamin D action are immune system cells. There, this vitamin can reduce the hypersecretion of chemokines and cytokines in monocytes [78], and control functions, maturation and/or growth of dendritic cells [79], T [80] and B cells [81]. A detailed review on the subject was published by Baeke *et al*. [82], but, in general, published studies indicate that 1,25(OH)_2_D_3_ acts on various cells of the immune system to generate a more tolerant and anti-inflammatory response profile (Figure 2).

### 5.2. Scientific Evidences

Many observational studies have shown a consistent and strong negative association between obesity and/or T2DM indicators and vitamin D serum levels [83–96]. Nevertheless, some observational studies that have evaluated the effects of vitamin D on insulin secretion and/or action have shown mixed results, since the effects disappeared after adjustment for adiposity [97–100]. These results suggest that adiposity, and not the vitamin D serum levels, is what influences insulin.

Possible explanations for the lack of unanimity on the relationships between obesity, insulin action and secretion, and serum levels of vitamin D were described by Lamendola *et al*. [59]. According to the authors, the estimation (not direct measure) of the insulin-mediated glucose availability, the absence of methods for adjustments in large populational studies, and the possibility that low vitamin D levels are the consequence and not the cause of obesity are the main reasons for the divergence between some studies. However, even if obesity promotes the decline of circulating vitamin D levels, excess weight is a common condition in diabetic patients and this does not exclude a possible interference of vitamin D on insulin secretion and action. In other words, vitamin D supplementation may be effective in preventing or controlling T2DM even when low levels of this vitamin are due to adiposity, and this should be evaluated in intervention studies.

Randomized double-blind clinical trials that offer the best evidence on the cause and effect relationships between variables are still scarce and inconclusive to establish the relationship between vitamin D, obesity and/or T2DM (Table 2). There is a wide variation in the doses (from 200 to the equivalent of 24,000 IU/day) and intervention time (6 weeks to 7 years). The studies that have associated vitamin D and calcium used doses of 500 to 1500 mg/day of the mineral.

The effects of vitamin D, without caloric restriction, on weight/adiposity control were observed in two recent studies [101,102]. In the first study [101], adult men and women who received 300 IU of vitamin D daily in conjunction with 1050 mg of calcium had a 160% increase in visceral fat reduction compared to the placebo group. In the second [102], a negative association between changes in vitamin D serum levels and BMI was observed in elderly subjects after the daily intake of 600 IU of vitamin D. These results indicate that vitamin D, even at doses within the recommended ranges and when not associated with calcium or restrictive diet, can help reduce body fat and that individuals with lower baseline levels of the vitamin may be most benefited. Another study [103], however, showed no improvement in the success of the intervention with the addition of vitamin D in relation to the group that received only calcium. In this study [103], postmenopausal women gained less truncal fat and more lean mass remained in the same region when they received 1100 IU vitamin D combined with 1400 to 1500 mg calcium or just the same dose of calcium daily for four years. It should be mentioned that the calcium doses adopted were higher than current recommendations and that, at recommended doses, the effects of the increased intake of vitamin D may be more easily detected.

When coupled with nutritional interventions aimed at weight loss, vitamin D appears to improve treatment response in relation to lipid profile [104] and serum PTH levels, triglycerides and TNF-α inflammation marker [105], but not to the adiposity markers [104,105]. A good lipid profile and inflammatory status reduction are essential for healthy weight loss, since the occurrence of transient dyslipidemia during energy restriction diets is common, and that, along with inflammation resulting from excess weight, may increase the risk of developing cardiovascular disease.

The results of two studies showed no positive effects of vitamin D on obesity markers [106,107]. These studies used higher doses than those previously cited (2857 to 24,000 IU/day) and one of them [106] lasted only 6 weeks. Three months of vitamin D supplementation are needed to achieve adequate serum levels for those deficient [13,107]. It is possible that very high doses of vitamin D may impair its function, as was evidenced in studies that evaluated the effect of high doses of vitamin D on bone health, and a minimum duration of 3 months is required in studies that assess the effects of supplementation of this vitamin.

Despite the short duration of the study conducted by Nagpal *et al*. [106], it was possible to observe an improvement in postprandial insulin sensitivity with the use of 360,000 IU of vitamin D every two weeks (24,000 IU/day) for six weeks. Pittas *et al*. [84] observed that the use of moderate doses of this vitamin (700 IU/day) associated with calcium over 3 years promoted a lower increase in fasting glucose and the HOMA-IR index in older adults with altered fasting glucose, but not in those with normal fasting glucose. Vitamin D can thus improve glycemic control and decrease insulin resistance, especially in subjects with altered glucose, contributing to the prevention and treatment of T2DM. Nevertheless, a robust study conducted with 33,951 postmenopausal women [85] showed no protective effect of a dietary intake of 400 IU of vitamin D combined with 1000 mg of calcium in reducing the incidence of T2DM. The effects of different doses of Vitamin D, alone or in conjunction with calcium on the prevention and/or treatment of T2DM need to be elucidated.

## 6. Conclusions

Vitamin D has hormonal action on various tissues and organs and its functions have been intensively reassessed with the discovery of the vitamin D receptor in most cells of the human body. The effects of vitamin D on bone metabolism are the best established. Besides increasing the circulating levels of calcium and phosphorus and promoting the mineralization process, this vitamin controls osteoblast and osteoclast function/differentiation and promotes bone formation, possibly by mechanisms to combat parathyroid hormone hypersecretion. These effects are evident in most intervention studies, especially in those that have evaluated the effects of vitamin doses equal to or higher than 800 IU/day on bone mineral density and that include calcium. Individual characteristics influence the response to the vitamin, such as the presence of obesity, which can increase the vitamin D demand, and the type of polymorphism of the vitamin D receptor. However, the use of megadoses of vitamin D with large intervals between administrations should be avoided, as they impair bone health.

Vitamin D seems to contribute to obesity and type 2 diabetes control by several mechanisms, including the regulation of adipogenesis during adipocyte differentiation, the stimulation of insulin synthesis and protection of pancreatic β-cells, and reducing insulin resistance in muscles. This vitamin may also contribute indirectly to combat these diseases through its action on bone tissue and the immune system, which liberates mediators that influence body weight gain and the inflammatory state. Nevertheless, recent studies question the cause and effect relationship between serum vitamin D levels and obesity. To date, there is not enough scientific evidence to support the use of vitamin D as a way to prevent and/or treat obesity and diabetes. Performing double-blind large-scale controlled trials to better establish the effects of vitamin D in chronic diseases of non-bone origin are required.

## Figures and Tables

**Table 1.** Effects of vitamin D supplementation with or without calcium on serum vitamin D and bone status.

Study	n (gender) y, duration	Vit D suppl.	Ca^++^ suppl.	Design	Final serum 25(OH)D_3_ (nmol/L)	Bone outcomes
Trivedi *et al*. [47]	2686 (649F, 2037M) 65–85 y, 5 years	100,000 IU/4 months	-	- Oral vit D - Control	- Vit D: 74.3 - Control: 53.4	- RR (treatment *vs*. control) of 0.78 (95% CI 0.61–0.99) for any first fracture, 0.67 (0.48–0.93) for first hip, wrist or forearm, or vertebral fracture
Harwood *et al*. [33]	150 (F) 67–92 y, One year	800 IU/day; 300,000/year	1000 mg/day	- Single injection - Injection + oral Ca^++^ - Oral vit D + oral Ca^++^ - Control	- Treatment groups: 40.0–50.0 - Control: 27.0	- ↑ BMD (control *vs*. treatments): 1.1%–3.3% neck of femur; 2.5%–4.6% trochanter; 2.1%–4.6% total hip. - RR of fall: 0.48 (95% CI 0.26–0.90)
Larsen *et al*. [48]	9605 (5771F, 3834M) 66–103 y, 3 years	400 IU/day	1000 mg/day	- Oral vit D + oral Ca^++^ - Environmental and Health Program - Both programs - Control	- Vit D + Ca^++^: 47.0 * - Control: 38.0 *	- RR (treatment vit D + Ca^++^ *vs*. control) of 0.84 (95% CI 0.72–0.98) for fracture incidence
Grant *et al*. [49]	5292 (4481F, 811M) 70 y or older, 2–5.2 years	800 IU/day	1000 mg/day	- Oral vit D - Oral Ca^++^ - Oral vit D + oral Ca^++^ - Control	-	- Incidence of new fractures: NS
Porthouse *et al*. [50]	3314 (F) 70 y or older, 1.5–3.5 years	800 IU/day	1000 mg/day	- Oral vit D + oral Ca^++^ - Control	-	- Incidence of fractures: NS
Flicker *et al*. [51]	625 (593F, 32M) 2 years	10,000 IU/week (1); 1000 IU/day (2)	600 mg/day	- Oral vit D (start in 1 and finish in 2) + oral Ca^++^ - Oral Ca^++^ (control)	-	- Incident rate ratio (treatment *vs*. control) of 0.73 for falling (95% CI 0.57–0.95) - In subjects who took at least half the prescribed capsules: Incident rate ratio of 0.63 (95% CI 0.48–0.82) for falls; OR of 0.70 (95% CI 0.50–0.99) for any falling
Jackson *et al*. [34]	36,282 (F) 50–79 y, 7 years	400 UI/day	1000 mg/day	- Oral vit D + oral Ca^++^ - Control	-	- ↑ BMD (treatment *vs*. control): 1.06% (*p <* 0.01); - HR for hip, clinical spine, or total fractures: NS
Lyons *et al*. [52]	3440 (2624F, 816M) 62–107 y, 3 years	100,000 IU/4 months	-	- Oral vit D - Control	- Vit D: 80.1 - Control: 54.0	- Incidence of fractures: NS
Smith *et al*. [53]	9440 (5086F, 4354M) 75 y or older, 3 years	300,000 IU/year	-	- Intramuscular vit D - Control	-	- HR (treatment *vs*. control) of 1.49 for hip fracture (95% CI 1.02–2.18) - HR for any first fracture or for wrist: NS
Zhu *et al*. [26]	120 (F) 70–80 y, 5 years	1000 IU/day	1200 mg/day	- Oral vit D + oral Ca^++^ - Oral Ca^++^ - Control	- Vit D + Ca^++^: 106.4 ± 29.0 - Ca^++^: 63.7 ± 28.0 - Control: 61.5 ± 23.0	- Ca^++^ and Vit D + Ca^++^ groups: maintenance of hip BMD *vs*. control group at year 1 - Only Vit D + Ca^++^ group keeps this result at year 3 (2.8% ± 1.1%, *p =* 0.01) and 5 (2.2% ± 1.1%, *p =* 0.05) - More pronounced results in individuals with less 25(OH)D_3_ at baseline.
Pfeifer *et al*. [54]	242 (191F, 51M) 70 y or older, One year	800 IU/day	1000 mg/day	- Oral Ca^++^ - Oral vit D + oral Ca^++^	- Ca^++^: 57.0 - Vit D + Ca^++^: 84.0	- RR (treatment *vs*. control) of 0.73 for first falls (95% CI 0.54–0.96)
Kärkkäinen *et al*. [35]	593 (F) 66–71 y, 3 years	800 IU/day	1000 mg/day	- Oral vit D + oral Ca^++^ - Control	- Vit D + Ca^++^: 74.6 ± 21.9 - Control: 55.9 ± 21.8	- ↑ BMD (final *vs*. initial): 0.84% (treatment) *vs*. 0.19% (control) - BMD change differences at the lumbar spine, femoral neck, trochanter, and total proximal femur: NS
Moschonis *et al*. [36]	66 (F) 55–65 y, 2.5 years	300 IU/day (1) 900 IU (2)	1200 mg/day	- Oral vit D(1) + oral Ca^++^ for 1 y and oral vit D(2) + oral Ca^++^ for 1.5 y - Control	-	- Changes (final–initial; control *vs*. treatment): BMD in arms: −0.047; 0.033, *p <* 0.001 BMD in total spine: 0.049; 0.118, *p =* 0.001; BMD in total body: −0.020; 0.003, *p <* 0.001
Jorde *et al*. [37]	421 (265F, 156M) 21–70 y, One year	40,000 IU/week (1) 20,000 IU/week (2)	500 mg/day	- Oral vit D(1) + oral Ca^++^ - Oral vit D(2) + oral Ca^++^ - Oral Ca^++^ (control)	- Vit D(1) + Ca^++^: 141.0 (1) - Vit D(2) + Ca^++^: 100.0 (2) - Ca^++^ (control): 57.9	- BMD at the lumbar spine and the hip: NS
Sanders *et al*. [55]	2256 (F) 70 y or older, 3–5 years	500,000 IU/year	-	- Oral vit D - Control	- Vit D: 74.0 - Control: ~50.0	- RR (treatment *vs*. control) of 1.15 for fell (95% CI 1.02–1.30) - RR (treatment *vs*. control) for fracture was 1.26 (1.00–1.59) - A temporal pattern was observed for falls (RR of 1.31 in the first 3 months and 1.13 during the following 9 months)
Salovaara *et al*. [56]	3432 (F) 65–71 y, 3 years	800 IU/day	1000 mg/day	- Oral vit D + oral Ca^++^ - Control	-Vit D + Ca^++^: 74.6 - Control: 55.9	- HR for any first fracture, nonvertebral, distal forearm or upper extremity fractures: NS
Islam *et al*. [38]	200 (F) 16–36 y, One year	400 IU/day	600 mg/day	- Oral vit D(VD) - Oral vit D + oral Ca^++^ (DC) - Oral vit D + oral Ca^++^ + micronutrients (VDCM) - Control (C)	- VD: 69.2 - VDC: 70.2 - VDCM: 64.8 - C: 35.5	- Changes (final–initial; control *vs*. treatments) BMD in femoral neck: −0.010 (C); 0.012 (VD); 0.013 (VDC); 0.017 (VDCM); *p <* 0.001; BMD in trochanter: −0.017 (C); 0.002 (VD); 0.001 (VDC); 0.010 (VDCM); *p <* 0.001
Rastelli *et al*. [39]	60 (F) Mean values of 60 and 63 y 6 months	400 IU/day + 50,000 IU/weekly and then monthly	1000 mg/day	- Oral vit D + oral Ca^++^ - Oral vit D (400 IU/d) + oral Ca^++^ (control)	- Vit D + Ca^++^: 74.3 - Control: 63.8	- BMD at the femoral neck decreased in the placebo and did not change in the treatment group (*p =* 0.06)
Steffensen *et al*. [40]	71 (F, M) 18–50 y, 2 years	20,000 IU/week	500 mg/day	- Oral vit D + oral Ca^++^ - Oral Ca^++^ (control)	- Vit D + Ca^++^: 123.2 - Control: 61.8	- BMD did not differ between groups at total hip, lumbar spine, and ultra-distal radius
Verschueren *et al*. [41]	113 (F) 70 y or older, 6 months	880 IU/day (1) 1600 IU/day (2)	1000 mg/day	- Oral vit D(1) + oral Ca^++ **^ - Oral vit D(2) + oral Ca^++ **^	- Vit D(1) + Ca^++^: 77.6 - Vit D(2) + Ca^++^: 84.6	- High dose of vitamin D did result in higher serum vitamin D levels but did not result in hip BMD improvements
Grimnes *et al*. [42]	297 (F) 50–80 y, One year	6500 IU/day (1) 800 IU/day (2)	1000 mg/day	- Oral vit D(1) + oral Ca^++^ - Oral vit D(2) + oral Ca^++^	- Vit D(1) + Ca^++^: 185.4 - Vit D(2) + Ca^++^: 89.2	- BMD was unchanged or slightly improved with no significant differences between the groups - Vit D(2) may be more efficient in reducing bone turnover
Nieves *et al*. [43]	103 (F) Mean values of 62.3 and 61.2 y, 2 years	1000 IU/day	1000 mg/day	- Oral vit D + oral Ca^++^ - Oral Ca^++^ (control)	- Vit D + Ca^++^: ~55.0 - Control: ~31.2	- Changes in BMD were not different between placebo- and vitamin D-treated black women at lumbar spine, total hip, and femoral neck - Femoral neck BMD was only responsive to vitamin D in VDR Fok1 polymorphism FF subjects, not Ff/ff subjects
Macdonald *et al*. [44]	305 (F) 60–70 y, One year	400 IU/day (1) 1000 IU/day (2)	-	- Oral vit D(1) - Oral vit D(2) - Control	- Vit D(1): 76.4 - Vit D(2): 65.4 - Control: 29.7	- BMD loss at the hip was less for the 1000 IU vitamin D group (0.05%) compared with the 400 IU vitamin D or placebo groups (0.57% and 0.60%, respectively) (*p <* 0.05)
Wamberg *et al*. [45]	52 (F, M) 18–50 y, 6 months	7000 IU/day	-	- Oral vit D - Control	- Vit D: 110.0 - Control: 46.8	- BMD at the ultradistal forearm significantly increased in the treatment group compared with a decrease in the placebo group - Changes in BMD between groups not differ at lumbar spine, hip or whole body

BMD: bone mineral density; Y: years; Vit D: vitamin D; Ca: calcium; CI: confidence interval; HR: hazard ratio; RR: relative risk; OR: odds ratio; NS: nonsignificant;

* After 2 years;

** With or without Whole-Body Vibration Training Program.

**Table 2.** Effects of vitamin D supplementation with or without calcium on obesity and diabetes parameters.

Study	n (gender) y, duration	Vit D suppl.	Ca^++^ suppl.	Design	Outcomes
Major *et al*. [104]	63 (F) ~42.6 y, 15 weeks	200 IU/day	600 mg/day	- Double-blind RCT - Oral vit D + oral Ca^++^ + WRP - Placebo + WRP	- Greater decreases (treatment *vs*. control) in total cholesterol/LDL, LDL/HDL and LDL - The differences were independent of changes in fat mass and in WC - Nonsignificant effects on BMI, fat mass or WC
Pittas *et al*. [84]	314 (F, M) 65 y or older, 3 years	700 IU/day	500 mg/day	- Double-blind RCT - Oral vit D + oral Ca^++^ - Placebo	- Participants with IFG: treatment group had a lower rise in fasting glucose compared with those on placebo and a lower increase in HOMA-IR - These differences were not present in normal fasting glucose subjects - There were no differences in C-reactive protein or IL-6 between groups
de Boer *et al*. [85]	33,951 (F) 50–79 y, 7 years	400 IU/day	1000 mg/day	- Double-blind RCT - Oral vit D + oral Ca^++^ - Placebo	- No significant results of dietary treatment on HR for incident diabetes - This null result was robust in subgroup analyses, efficacy analyses accounting for nonadherence, and analyses examining change in laboratory measurements
Nagpal *et al*. [106]	100 (M) 35 y or older, 6 weeks	360,000 IU/fortnightly	-	- Double-blind RCT - Oral vit D - Placebo	- Increase in postprandial insulin sensitivity in treatment - No changes in secondary outcome (insulin secretion, basal indices of insulin sensitivity, blood pressure or lipid profile) were found
Sneve *et al*. [107]	445 (F, M) 21–70 y, 12 months	20,000 IU/twice a week (1) 20,000 IU/once a week (2)	500 mg/day	- Double-blind RCT - Oral vit D(1) - Oral vit D(2) + placebo - Placebo	- No significant change in weight, waist-to-hip ratio or % body fat - PTH decrease and 25(OH)D_3_ increase in treatments groups, and 25(OH)D_3_ stabilized after 3 months
Zitterman *et al*. [105]	200 (F, M) 18–70 y, 12 months	3320 IU/day	-	- Double-blind RCT - Oral vit D + WRP - Placebo + WRP	- Weight loss was not affected significantly - More pronounced decrease occurred in treatment group than in the placebo group in PTH, triglycerides, and the inflammation marker TNF-α. Vitamin D increased LDL
Zhou *et al*. [103]	870 (F) 55 y or older, 4 years	1100 IU/day	1400–1500 mg/day	- Double-blind RCT - Placebo + oral Ca^++^ - Oral vit D + oral Ca^++^ - Placebo	- The calcium intervention groups gained less trunk fat and maintained more trunk lean mass when compared to the placebo group, without difference with adding vitamin D - No significant difference was observed for BMI between groups
Rosenmblum *et al*. [101]	171 (F, M) 18–65 y, 16 weeks	300 IU/day	1050 mg/day	- Double-blind RCT - Oral vit D + oral Ca^++^ - Placebo	- Treatment group increase decrease significantly more the % of visceral adipose tissue (−13 ± 16 *vs*. −5 ± 19)
Forsythe *et al*. [102]	212 (F, M) 20–40; >64 y, 22 weeks	600 IU/day	-	- RCT - Oral vit D - Placebo	- BMI in older adults was negatively associated with the change in 25(OH)D following supplementation. No such associations were apparent in younger adults

RCT: randomized controlled trial; HR: hazard ratio; IFG: impaired fasting glucose; WRP: weight reduction program; BMI: body mass index; WC: waist circumference.
